# Comparison of VO_2_max Estimations for Maximal and Submaximal Exercise Tests in Apparently Healthy Adults

**DOI:** 10.3390/sports11120235

**Published:** 2023-11-27

**Authors:** Marc-Olivier Dugas, Pénélope Paradis-Deschênes, Laurie Simard, Tommy Chevrette, Patricia Blackburn, Martin Lavallière

**Affiliations:** 1 Unité D’enseignement en Kinésiologie, Département des Sciences de la Santé, Université du Québec à Chicoutimi (UQAC), Saguenay, QC G7H 2B1, Canada; 2 Laboratoire de Recherche Biomécanique & Neurophysiologique en Réadaptation Neuro-Musculo-Squelettique—Lab BioNR, Université du Québec à Chicoutimi (UQAC), Saguenay, QC G7H 2B1, Canada; 3 Centre Intersectoriel en Santé Durable (CISD), Université du Québec à Chicoutimi (UQAC), Saguenay, QC G7H 2B1, Canada; 4 Observatoire du Développement Moteur et Psychomoteur des 0-18 ans, Saguenay, QC G7H 2B1, Canada

**Keywords:** VO_2_max, exercise test, treadmill, metabolic equation, ACSM, cardiorespiratory fitness

## Abstract

Due to limited accessibility, direct measurement of VO_2_max is rarely performed in clinical settings or sports centers. As a result, regression equations have been developed and are currently used during exercise tests to provide an indirect estimation. The American College of Sports Medicine (ACSM) has recommended a regression equation for running to provide an indirect estimation of VO_2_. However, significant differences have been observed between these estimations and directly measured VO_2_max. Additionally, since submaximal assessments may be more convenient for both athletes and sedentary/diseased individuals, they were included in the analysis. This study aimed to evaluate the accuracy of VO_2_max estimations provided using the ACSM running equation when used during both maximal and submaximal exercise tests among adult runners. A total of 99 apparently healthy and active adults (age: 39.9 ± 12.2 years; VO_2_max: 47.4 ± 6.0 mL O_2_/kg∙min^−1^) participated in this study. Two types of submaximal estimations were performed to predict VO_2_max: one based on age-predicted maximal heart rate (HRmax) (ACSM_submax,Fox_), and the second using the actual HRmax measured during the exercise test (ACSM_submax,measured_). The measured VO_2_max was compared to these estimations obtained from a single exercise test. Both maximal and submaximal exercise tests significantly overestimated VO_2_max (ACSM_max_: +9.8, *p* < 0.001; ACSM_submax,Fox_: +3.4, *p* < 0.001; ACSM_submax,measured_: +3.8 mL O_2_/kg∙min^−1^, *p* < 0.001). However, the submaximal estimations were closer to the measured VO_2_max (*p* < 0.001). This analysis demonstrated that the included methods overestimated the true VO_2_max. Nonetheless, the submaximal exercise tests provided a more accurate prediction of VO_2_max compared to the maximal exercise tests when using the ACSM running equation.

## 1. Introduction

The highest rate of oxygen consumed by the body during exercise (VO_2_max) is frequently used to assess cardiorespiratory fitness because it is considered as its best simple measure [1,2]. It can be defined as the maximum amount of oxygen utilized by the body during strenuous exercise [1]. The work of Wasserman [3] illustrates the functional interdependence between the physiological components responsible for the aerobic system, namely the cardiovascular, pulmonary, and muscular system. The literature demonstrates a positive relationship between VO_2_max and a range of sport performances [1,4,5,6]. Additionally, extensive evidence suggests that low VO_2_max is a strong independent predictor of cardiovascular disease and premature death, showing that assessing VO_2_max is of significant interest for most individuals [7].

However, the gold standard assessment of VO_2_max is costly and requires the expertise of highly trained personnel. This method involves direct measurement of pulmonary gas and requires the participants to engage in a maximal exercise test until exhaustion. To improve accessibility, several VO_2_max prediction models have been developed. Some even eliminate the need for exercise testing, but yield inconsistent results [8]. On the other hand, indirect estimations of VO_2_max commonly involve maximal or submaximal exercise tests without direct pulmonary gas measurement. Although, evidence suggests that the risk of adverse events in maximal exercise tests are minimal under appropriate supervision [9]; submaximal exercise tests are generally considered safer and more convenient options, especially for sedentary populations or individuals with chronic health conditions since they can eliminate the need to obtain medical approval, as suggested with common exercise preparticipation health screening algorithms [10].

The American College of Sports Medicine (ACSM) presents widely used and globally recognized guidelines [10]. Their regression equation for running estimates the required VO_2_ to sustain a physical exertion based on speed and incline [10]. This equation can be applied in various evaluation settings and with different protocols. However, the validity of this equation is questionable as, to the best of our knowledge, no study has yet demonstrated its accuracy. On the contrary, most studies on the subject have identified overestimations in VO_2_ and VO_2_max using the equation [11,12,13] and it appears to be based on a small and non-representative sample of the general population [11,14,15]. For instance, three studies performed with groups of apparently healthy and active males found significant VO_2_max overestimations ranging from 9.5 to 18.0% [12,16,17]. Furthermore, to the best of our knowledge, no study has compared both maximal and submaximal VO_2_max estimations using this equation.

The objective of this study was to validate the estimations of VO_2_max using the ACSM running equation in apparently healthy active runners, during both maximal and submaximal exercise tests. For comparison, VO_2_max was measured using a metabolic analyzer. Our secondary aim was to determine whether the ACSM maximal exercise tests offer a more accurate VO_2_max estimation compared to the ACSM submaximal exercise tests. Based on our observations, we believe that the ACSM running equation will overestimate the VO_2_max, and that the submaximal exercise tests will be closer to the actual VO_2_max measured through a metabolic cart.

## 2. Materials and Methods

### 2.1. Participants

This cross-sectional study involved a total of 99 apparently healthy active adults (58 men and 41 women) who were evaluated. Participants were recruited from the University Sports Center and local running clubs in the Saguenay–Lac-Saint-Jean area. The inclusion criteria were being in good health, running at least twice a week for more than 6 weeks, and being able to run continuously for 30 min. Individuals with any disease or injury affecting running or consuming medications that affect heart rate (HR) were excluded. All participants provided their consent using an electronic form prior to testing. The study was approved by the Université du Québec à Chicoutimi Ethical Research Committee (#2019-1).

All participants were requested to follow these guidelines prior to testing: 2 h without eating and smoking, 48 h without alcohol and 12 h without beverages containing caffeine, 24 h without exercise, 24 h without consuming natural products and medicines for a cold, a cough, or asthma or pain relievers, and finally, we also asked the participants to obtain 3 days of normal sleep (the same sleep they normally obtain). Each participant was told to be hydrated prior to testing (drink approximately 500 mL of water 2 h prior to testing).

### 2.2. Protocol

All tests were performed in the same testing laboratory (ambient temperature: 20 °C, humidity: 45–55%) and treadmill (770T, Cybex, Franklin Park, IL, USA), which was previously calibrated for speed and slope. Participants attended a single 1 h visit to the laboratory to complete the exercise test. Participants arrived at the testing laboratory between 2:00 and 7:30 P.M. Upon arrival, evaluation procedures were explained verbally, and any questions were addressed. HR was measured at the radial pulse for 15 s, and blood pressure was measured using a sphygmomanometer (Physio Logic, AMGMedical, Mont-Royal, QC, Canada). Body mass (700, Seca, Hamburg, Germany) and body height (213, Seca, Saulieu, France) were measured to the nearest 0.1 kg and cm, respectively, following the recommendations of the Canadian Society for Exercise Physiology [18].

The “Combiné-Drolet” test protocol was used to obtain indirect and direct VO_2_max measurements. This step-incremented exercise protocol offered the advantage of calculating indirect VO_2_max using the last completed stage of the maximal exercise test, and extrapolating the results of the first three stages of the submaximal exercise test, all performed in a single session. This approach aligns with the ACSM’s recommendations for both a maximal and submaximal exercise test [19]. The exercise tests were either conducted or supervised by a certified and experienced kinesiologist, with the assistance of a kinesiology student. After a 3 min warm-up, participants ran during three levels that were the same for everyone (level 1: 8.0 km/h and 0% incline; level 2: 9.0 km/h and 1% incline; level 3: 10.0 km/h and 2% incline). These levels lasted 3 min and allowed for the submaximal evaluation of VO_2_max. Subsequent levels lasted 2 min each, with speed and incline increments adjusted according to the participants’ physical capacity until they reached physical exhaustion. To determine appropriate speed and grade increments, known running times for a 5 or 10 km distance were commonly used. Essentially, by increasing the running speed by approximately 15% based on the participant’s current best-known 5 or 10 km pace, we were able to reach their voluntary termination around the 5th stage or during the 6th stage. This, including the submaximal part of the test, corresponds to a test duration of approximately 13 to 15 min on average. All participants ran until voluntary termination of the exercise test. Moreover, to confirm that VO_2_max was reached during the test, we ensured that all the following criteria were attained: HR ≥ 85% of predicted HRmax; respiratory exchange ratio ≥1.1, and/or a plateau of VO_2_ (ΔVO_2_ < 150 mL O_2_∙min^−1^); participants could not continue despite encouragement and had a rating of perceived exertion (RPE) > 7/10 [19,20,21]. For participants who did not meet all these criteria, the test was either repeated on another day or they were excluded from the analyses.

Direct measurement of VO_2_max using a pulmonary gas analysis was conducted using a Vyntus CPX metabolic analyzer (Jaeger-CareFusion, Höchberg, Germany) during the exercise test. The software calculated the average of all the ergospirometric data based on the last 8 complete respiration cycles. The results were compared to the estimates to assess their precision. This metabolic cart has been shown to be valid and reliable [22,23]. Before testing, the gas analyzer was calibrated according to the manufacturer’s instructions using a certified standard mixture of oxygen (16%) and carbon dioxide (4%), balanced with nitrogen. HR was continuously measured during the test using an HR monitor with a chest strap (RS800, Polar, Kempele, Finland).

Indirect estimation of VO_2_max was obtained using the ACSM running equation (VO_2_ in ml O_2_/kg∙min^−1^ = (0.2 speed (meter/minute)) + (0.9 speed (meter/minute) fractional grade) + 3.5) in both the maximal and submaximal parts of the exercise test. For the maximal part, the last completed stage of the test (ACSM_max_) was used. For the submaximal part, a regression equation was created using the mean HR during the last 15 s of the first three levels, with VO_2_ (estimated using the same ACSM running equation) on the *x*-axis and HR on the *y*-axis. Using the HRmax, the equation was extrapolated to find x, which represented VO_2_max. Two HRmax values were used: one age-predicted (220-age) using the Fox equation (ACSM_submax,Fox_) [24], and the other measured during the maximal exercise test (ACSM_submax,measured_). It is worth noting that in one instance, the chest strap fell, which prevented the measurement of HRmax. Therefore, there was one fewer participant in the ACSM_submax,measured_ group compared to the others.

All submaximal exercise tests followed the criteria outlined in the ACSM statement [19] (3): steady HR attainment on each level (±5 bpm between the end of the 2nd and 3rd minute) or a 1-min extension was added to the level; attainment of ≥70% reserve HR or ≥85% age-predicted HRmax ± 5 bpm (Fox equation).

To reduce the risk of an outlier for HR, the HR used for extrapolations and test validation was a mean calculated from three HR measurements at 15, 10, and 5 s before the end of the level.

### 2.3. Statistical Analysis

Data are presented as the mean ± standard deviation (SD). Statistical analyses were performed using SPSS statistics software for Windows version 26 (Chicago, IL, USA). A *p*-value ≤ 0.05 was considered statistically significant. An ANCOVA with random effects of the individual was performed to compare the estimation methods and direct VO_2_max measurements while controlling for confounding variables. Residuals were tested for normal distribution using the Shapiro–Wilk test. Homogeneity of residual variance was assessed with a graph plotting fixed predictions on the *y*-axis and residuals on the *x*-axis. Descriptive data were generated for age, body height, body mass, BMI, speed and incline of the last completed stage, HRmax, and VO_2_max with Microsoft Excel 2016 (Redmond, WA, USA). Bland–Altman plots, and the mean absolute percentage error (MAPE), were also created and calculated using Microsoft Excel. A sample size of 98 achieves 80% power to detect an effect size of 0.286, with an estimated standard deviation of differences of 1.00, and a significance level (alpha) of 0.05 using a two-sided paired *t*-test.

## 3. Results

A total of 99 exercise tests were completed and analyzed. Therefore, the current dataset includes 99 participants from 19 to 66 years old (age: 39.9 ± 12.2 years) with normal body mass (BMI: 23.6 ± 2.8 kg/m^2^). The participants exhibited a range of VO_2_max values from 34.8 to 63.0 mL O_2_/kg∙min^−1^, with an average VO_2_max of 47.4 ± 6.0 mL O_2_/kg∙min^−1^. Table 1 presents physical characteristics of the subjects and the data obtained during the exercise tests.

Table 2 provides the adjusted means and 95% confidence intervals (CIs) for all the estimates included in this study, as well as the measured VO_2_max, accounting for confounding variables.

Table 3 displays the comparisons with Bonferroni adjustment between the VO_2_max estimates and the measured VO_2_max, revealing that all methods significantly overestimated VO_2_max. The maximal exercise tests (ACSM_max_) exhibited the highest overestimation, with an average of 9.8 mL O_2_/kg∙min^−1^. Furthermore, the submaximal methods also significantly overestimated VO_2_max, with 6.4 (ACSM_submax,Fox_) and 6.0 mL O_2_/kg∙min^−1^ (ACSM_submax,measured_) on average.

The mean absolute percentage errors between the estimates and the measured VO_2_max (Vyntus) were 21.6% (ACSM_max_), 11.3% (ACSM_submax,Fox_), and 10.8% (ACSM_submax,measured_).

**Figure 1:**
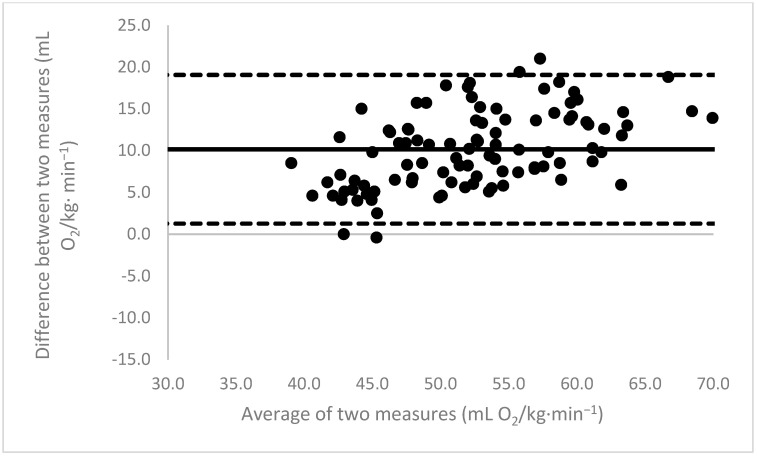
ACSM_max_ Bland–Altman plot (all). The solid line depicts the mean difference, and the dotted lines illustrate the limits of agreement.

**Figure 2:**
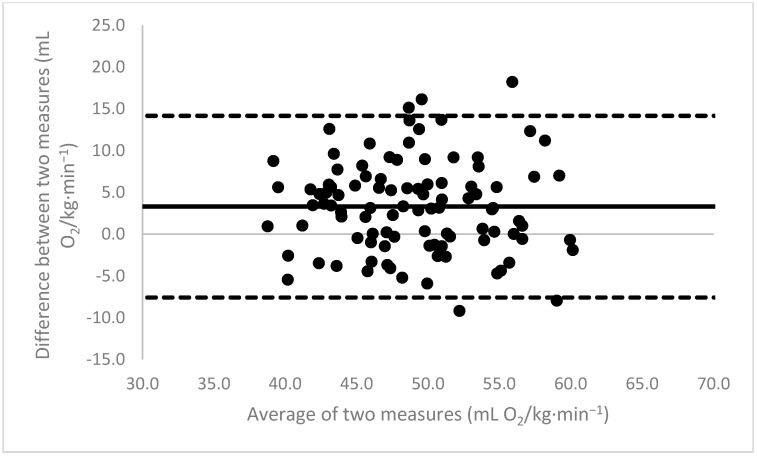
ACSM_submax,Fox_ Bland–Altman plot (all). The solid line depicts the mean difference, and the dotted lines illustrate the limits of agreement.

**Figure 3:**
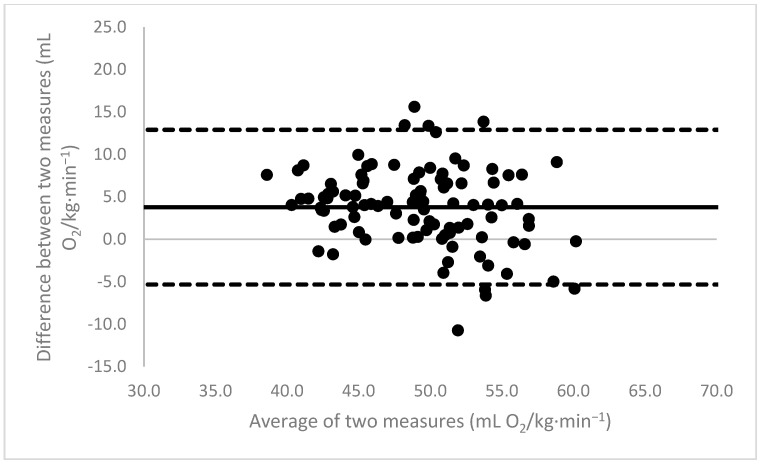
ACSM_submax,measured_ Bland–Altman plot (all). The solid line depicts the mean difference, and the dotted lines illustrate the limits of agreement.

**Figure 4:**
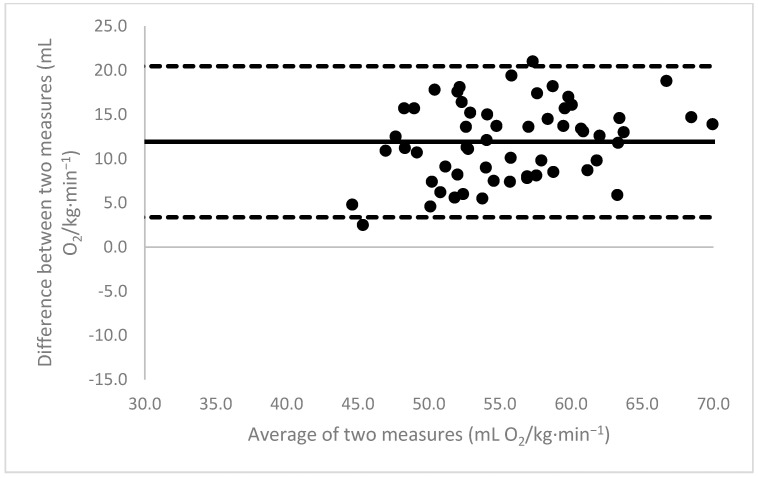
ACSM_max_ Bland–Altman plot (men). The solid line depicts the mean difference, and the dotted lines illustrate the limits of agreement.

**Figure 5:**
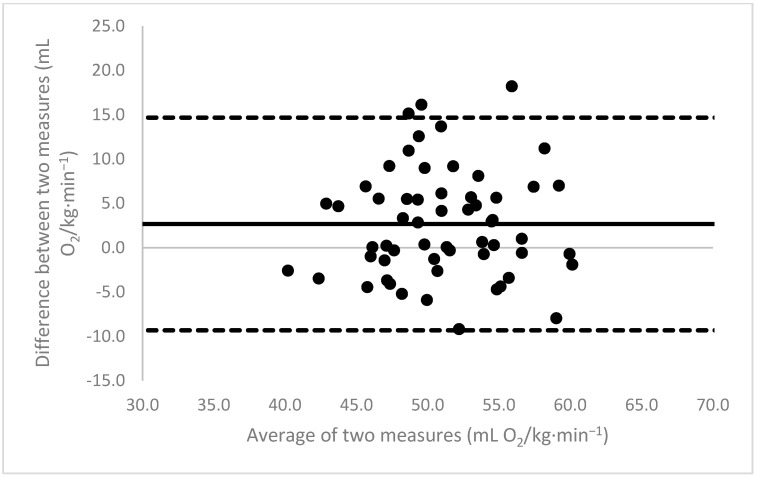
ACSM_submax,Fox_ Bland–Altman plot (men). The solid line depicts the mean difference, and the dotted lines illustrate the limits of agreement.

**Figure 6:**
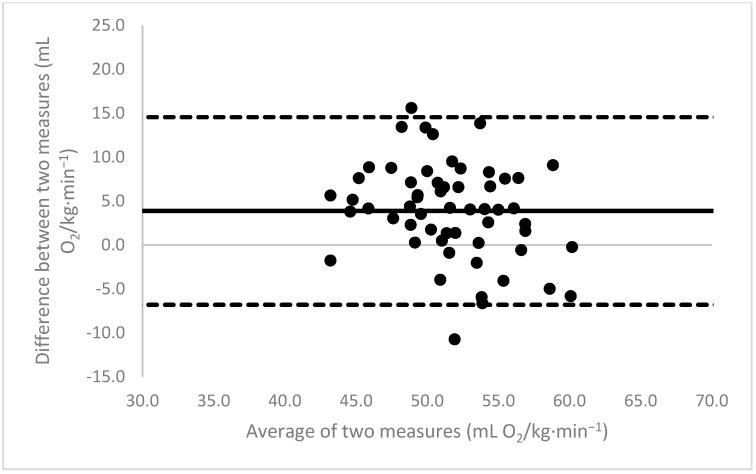
ACSM_submax,measured_ Bland–Altman plot (men). The solid line depicts the mean difference, and the dotted lines illustrate the limits of agreement.

**Figure 7:**
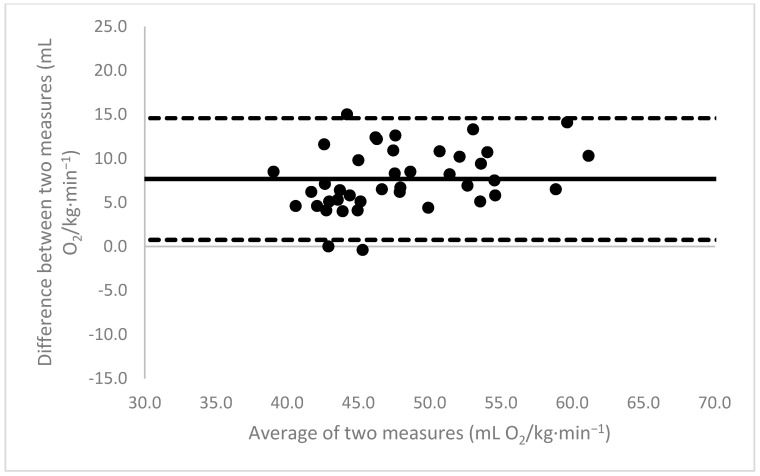
ACSM_max_ Bland–Altman plot (women). The solid line depicts the mean difference, and the dotted lines illustrate the limits of agreement.

**Figure 8:**
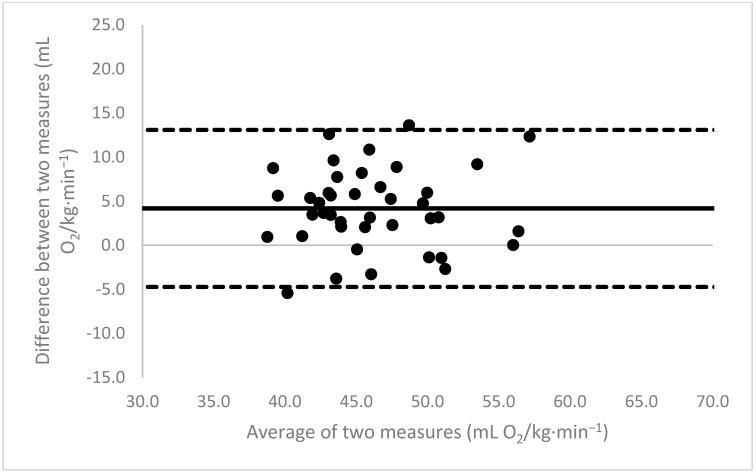
ACSM_submax,Fox_ Bland–Altman plot (women). The solid line depicts the mean difference, and the dotted lines illustrate the limits of agreement.

**Figure 9:**
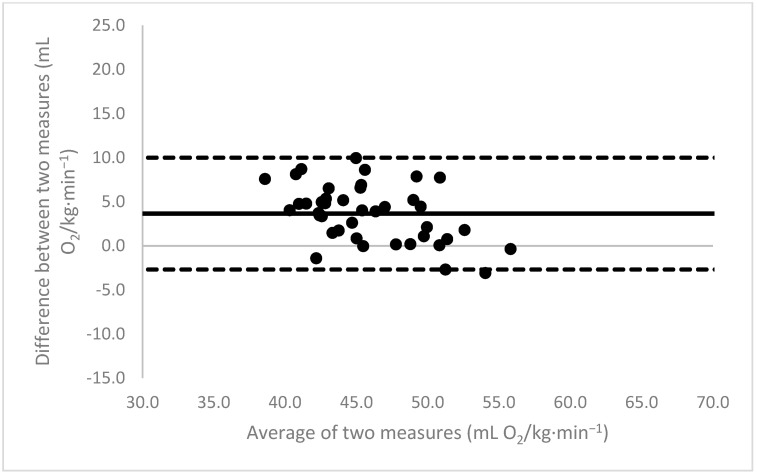
ACSM_submax,measured_ Bland–Altman plot (women). The solid line depicts the mean difference, and the dotted lines illustrate the limits of agreement.

Figure 1, Figure 2, Figure 3, Figure 4, Figure 5, Figure 6, Figure 7, Figure 8 and Figure 9 depict Bland–Altman plots illustrating the differences between the measured VO_2_max and the estimates using ACSM_max_, ACSM_submax,Fox_, and ACSM_submax,measured_ for the entire cohort (Figure 1, Figure 2 and Figure 3), men (Figure 4, Figure 5 and Figure 6), and women (Figure 7, Figure 8 and Figure 9), respectively (in ml O_2_/kg∙min^−1^). The *x*-axis represents the mean between the measured and estimated VO_2_max for each individual, while the *y*-axis displays the difference between the two for each individual. The solid line represents the mean difference, and the dotted lines represent the limits of agreement. These plots support the analysis by demonstrating, on average, a larger bias in the ACSM_max_ compared to ACSM_submax,Fox_ and the ACSM_submax,measured_. For example, they not only highlight a higher mean bias but also that the lower limit of agreement is greater than the measured VO_2_max value, unlike the submaximal methods.

## 4. Discussion

The objective of this study was to compare the estimations of VO_2_max for maximal and submaximal exercise tests using the ACSM running equation. As expected, both the submaximal and maximal methods using the ACSM running equation overestimated VO_2_max for apparently healthy active adults, regardless of biological sex. However, the primary finding of this study was that submaximal exercise tests provided more accurate estimates of VO_2_max compared to maximal exercise tests using the ACSM equation. To the best of our knowledge, it is the first study to assess this question.

As expected, the ACSM running equation significantly overestimated VO_2_max during an exercise test performed to physical exhaustion by 20.9% on average. This aligns with similar studies conducted on active and athletic individuals [12,16,17]. For example, Koutlianos, Dimitros [12] observed a significant overestimation of 14.6% with the ACSM equation in 55 male athletes aged 18 to 37 years using a Bruce protocol. Similarly, overestimations of 9.3% [16] and 18.0% [17] were reported in young and active males using a Ramp protocol. However, unlike our study, none of these studies included women in their groups. These findings are also consistent with studies conducted on apparently healthy and elderly or diseased individuals (including hypertension, fibromyalgia, osteoarthritis, and cardiovascular disease) who used the ACSM walking or running equations for VO_2_max estimations [11,14,15,25,26,27]. Additionally, Kokkinos, Kaminsky [11] found an average overestimation of 21.4% using both the ACSM running and walking equations in a large sample of 7983 healthy subjects evaluated using nine different protocols. However, they did not confirm whether these differences were significant. While there is a considerable body of evidence on maximal exercise tests using the ACSM equation, none of these studies were conducted specifically on a group of individuals who regularly engage in running.

Several factors could explain why the use of the ACSM running equation leads to inaccurate VO_2_max estimates during a maximal exercise test. It has been suggested that the ACSM equations were developed for steady-state exercise, whereas in this case, they are being used during an exercise test with an incremental speed and/or grade [14,25,27]. Additionally, Berry, Brubaker [14] highlighted that at high intensities, including maximal intensity, the contribution of the anaerobic system increases. Furthermore, as explained by Kokkinos, Kaminsky [11], achieving a steady state is influenced by age, health, and physical activity levels. Also, according to Kokkinos, Kaminsky [15], the ACSM equations were developed based on a relatively small sample size (<200) of young participants (19 to 26 years old) who underwent a submaximal exercise test on a treadmill to achieve a steady-state aerobic requirement. VO_2_max was then extrapolated based on the achieved VO_2_ levels during steady-state exercise [15]. Additionally, other studies have also mentioned that the equations are based on a small sample size [11,14], but it was not possible to find direct evidence regarding the exact composition of the sample used to develop the ACSM equations. Berry, Brubaker [14] further specify that the walking equation comprises three components, horizontal, vertical, and a resting oxygen requirement component, and that these components are based, at least in part, on small numbers of healthy young men. The horizontal and vertical components appear to be based on Dill [28]’s work with only 3 male subjects, and Balke and Ware [29]’s work with 500 male military and civilian personnel [14], respectively. In summary, as pointed out by Filardo, Silva [30], although the ACSM’s metabolic equations have been proposed for widespread use, they are based on studies that do not indicate such use.

The overestimation of VO_2_max with submaximal running exercise tests is consistent with previous studies, although only one study has examined this matter using the ACSM running equation. In this study, the ACSM running equation significantly overestimated VO_2_max during a submaximal exercise test by 7.3% (ACSM_submax,Fox_) and 8.1% (ACSM_submax,measured_) on average. Marsh [31] found an average overestimation of 3% compared to the reference value in 21 moderately well-trained young men. However, this difference was not significant. Additionally, Lee, Bassett Jr. [32] compared the VO_2_max measured using a Douglas bag during a Bruce protocol carried out to exhaustion with the VO_2_max estimated using the ACSM walking equation during a submaximal Bruce protocol in 48 people aged between 18 and 59 years without contraindications to exercise. They observed a significant overestimation of 4.6 mL O_2_/kg∙min^−1^ (10.0%) between the measured VO_2_max and that estimated using the ACSM walking equation. Both studies used the age-predicted maximal HR according to Fox’s equation to perform the extrapolation.

Therefore, the superiority of submaximal exercise tests over maximal exercise tests (7.3% and 8.1% vs. 20.9% overestimation) might be attributed to some of the factors explaining the inaccuracy of the ACSM equations for maximal exercise tests, such as the assumption of a steady state and the higher contribution of the anaerobic system. Additionally, Cunha, Catalao [33] found that the velocities defined using the ACSM metabolic equation overestimate energy expenditure, and other studies have shown overestimations using the ACSM running equation during submaximal steady-state intensities [13,30]. Although submaximal exercise tests provided more accurate results compared to maximal exercise tests, it is evident that the documented overestimation could impact the ability to accurately measure VO_2_max.

Among the identified limitations, some participants had little or no experience in running on a motorized treadmill, and wearing a mask during an exercise test to volitional exhaustion could cause discomfort. Additionally, certain pre-test criteria, such as guidelines regarding alcohol or coffee consumption, physical activity, or sleep, were recommended, and it is possible that these criteria were only partially met, despite questioning the participants on this matter. Finally, although conducting a verification phase could have enhanced our confidence in the measured VO_2_max results, it is worth noting that all the analyzed tests successfully met the criteria for VO_2_max attainment as described in Section 2.

Based on our results, it is evident that indirect estimations of VO_2_max exhibit imprecision for a group of healthy runners. Direct measurement remains the gold standard for assessing cardiorespiratory fitness and should be preferred for athletic or sportive individuals. Given the magnitude of these differences, they can significantly impact the quality of interventions, as exercise prescriptions should be individualized based on cardiorespiratory fitness. However, it is acknowledged that direct measurement is not always feasible. Therefore, considering the results, submaximal exercise tests using the ACSM running equation could be preferred over maximal exercise tests to assess VO_2_max in runners. As previously mentioned, this is, to our knowledge, the first study to compare these test modalities. For athletic populations, although maximal exercise tests may provide valuable information for exercise prescription and evaluation, submaximal exercise tests are easier to perform and may better align with training schedules. These findings have greater implications for sedentary and symptomatic populations. Considering their conditions, submaximal exercise tests are more suitable and potentially safer, and they may eliminate the need for medical clearance prior to this physical evaluation. Further research is needed to evaluate the accuracy of submaximal exercise tests using the ACSM equations with symptomatic and sedentary individuals. A valid and appropriate VO_2_max estimation for clinical settings is necessary to assess treatment effectiveness, improve cardiovascular risk stratification, and provide individualized exercise prescriptions. It represents a valuable tool for various health professionals to improve the quality of their services.

## Figures and Tables

**Table 1:** Group demographics.

N (% men)	99 (58.6)
Age (years)	39.9 ± 12.2
Body height (m)	1.71 ± 0.08
Body mass (kg)	69.3 ± 11.4
BMI (kg/m^2^)	23.6 ± 2.8
Speed at the last stage completed (km/h)	14.6 ± 2.1
Grade at the last stage completed (%)	2.3 ± 0.7
Age-predicted HRmax (bpm)	180.1 ± 12.1
HRmax measured (bpm) *	181.9 ± 11.6
Measured VO_2_max (ml O_2_/kg∙min^−1^)	47.4 ± 6.0

* *n* = 98.

**Table 2:** Mean VO_2_max adjusted for cofounding variables between methods.

Methods	Sex	Mean	95% CI	
			Lower	Upper
Measured VO_2_max	All	46.8	45.8	47.9
ACSM_max_		56.6	55.6	57.6
ACSM_submax,Fox_		50.3	49.3	51.3
ACSM_submax,measured_		50.6	49.6	51.7
Measured VO_2_max	Men	49.9	48.6	51.2
ACSM_max_		61.9	60.5	63.2
ACSM_submax,Fox_		52.6	51.2	53.9
ACSM_submax,measured_		53.6	52.3	54.9
Measured VO_2_max	Women	43.8	42.3	45.4
ACSM_max_		51.4	49.8	53.0
ACSM_submax,Fox_		48.0	46.4	49.6
ACSM_submax,measured_		47.7	46.1	49.3

Data are expressed in ml O_2_/kg∙min^−1^.

**Table 3:** Pairwise comparisons between the estimates and the true VO_2_max.

Methods	Pairwise Comparisons				
	Methods	Mean Difference	Mean Difference (%)	95% CI	
				Lower	Upper
ACSM_max_	Measured VO_2_max	+9.8 ^‡^	+20.9	+8.5	+11.0
ACSM_submax,Fox_	Measured VO_2_max	+3.4 ^‡^	+7.3	+2.2	+4.7
ACSM_submax,measured_	Measured VO_2_max	+3.8 ^‡^	+8.1	+2.5	+5.1
ACSM_submax,Fox_	ACSM_max_	−6.4 ^‡^	−11.3	−7.6	−5.1
ACSM_submax,measured_	ACSM_max_	−6.0 ^‡^	−10.6	−7.2	−4.7
ACSM_submax,Fox_	ACSM_submax,measured_	−0.4	−0.8	−1.6	+0.9

Data are expressed in mL O_2_/kg∙min^−1^; ^‡^ *p* ≤ 0.001.

## Data Availability

The supporting dataset is available upon request to the corresponding author.
